# Effects and Challenges of Nuclear Medicine: A Cross-Sectional Survey of Health Professionals’ Perceptions in Saudi Arabia

**DOI:** 10.7759/cureus.112194

**Published:** 2026-07-07

**Authors:** Kady H Althunayan, Sara M Alshahwan, Mohammed S Alghurab, Abdullah M Rajab, Ibrahim A Eljack, Abdullah A Alqarni, Fatimah A Alakroush, Nouf A Alalmaei, Haneen Abanmi, Suaad E Elnour

**Affiliations:** 1 Department of Medicine, College of Medicine, University of Bisha, Bisha, SAU; 2 Department of Diagnostic Radiology and Nuclear Medicine, Imam Abdulrahman Bin Faisal University, Dammam, SAU; 3 Department of Medical Imaging and Radiation Science, Imam Abdulrahman Bin Faisal University, Dammam, SAU; 4 Department of Nuclear Medicine Technology and Radiation Safety, National Blood and Cancer Centre, Riyadh, SAU; 5 Department of Family and Community Medicine, College of Medicine, University of Bisha, Bisha, SAU; 6 Department of Internal Medicine, College of Medicine, University of Bisha, Bisha, SAU; 7 Department of Medicine, College of Medicine, King Faisal University, Al-Ahsa, SAU; 8 Department of Medicine, Majmaah University, Al Majma'ah, SAU; 9 Department of Obstetrics and Gynecology, College of Medicine, University of Bisha, Bisha, SAU

**Keywords:** cross-sectional study, health professionals, nuclear medicine, occupational radiation exposure, radiation safety, radiopharmaceuticals, saudi arabia

## Abstract

Background: Nuclear medicine plays a pivotal role in diagnosis and treatment through the use of radiopharmaceuticals. It also presents significant health and safety challenges due to occupational exposure to ionizing radiation. As nuclear medicine continues to expand across Saudi Arabia, evaluating healthcare professionals' knowledge, training adequacy, adherence to safety protocols, and perceived risks is essential to ensuring sustainable and safe practice.

Objectives: The aim of this study was to determine the practical effects and challenges in nuclear medicine, assess knowledge and perceptions of radiation safety, evaluate occupational exposure doses, and explore institutional challenges among nuclear medicine professionals across Saudi Arabia.

Methods: This cross-sectional survey enrolled 110 nuclear medicine professionals from all five geographic regions of Saudi Arabia using a structured, self-administered questionnaire distributed via Google Forms (Google LLC, Mountain View, CA, USA). Data were analyzed using IBM SPSS Statistics software, version 26 (IBM Corp., Armonk, NY, USA), employing descriptive statistics and chi-square tests (p ≤ 0.05).

Results: Respondents were predominantly male (69.1%) and Saudi nationals (98.2%), with 58.2% working in government hospitals. While 70% expressed moderate-to-high confidence in radiation knowledge, only 45.5% were familiar with dose limits, and 22.8% reported insufficient training. Among those with monitoring data, 5.5% reported maximum annual exposures exceeding 10 millisieverts (mSv). Notably, 77.3% worked 40-50 hours weekly, and 47.3% reported high occupational stress.

Conclusion: Significant gaps persist in safety training, dose-limit familiarity, and protective measure adherence. Comprehensive strategies are needed to enhance safety culture, ensure continuous education, and enforce radiation protection protocols in nuclear medicine departments across Saudi Arabia.

## Introduction

Nuclear medicine is a rapidly evolving medical specialty that uses radioactive tracers, radiopharmaceuticals, to diagnose and treat a wide range of diseases. Unlike conventional radiology, nuclear medicine imaging provides functional and molecular information about organ systems, often detecting nuclear medicine disease at earlier stages than other diagnostic modalities [1]. Procedures such as positron emission tomography/computed tomography (PET/CT), single-photon emission computed tomography (SPECT/CT), myocardial perfusion imaging, bone scintigraphy, and radioiodine therapy constitute the cornerstone of modern nuclear medicine practice [2-10].

Nuclear medicine professionals are considered among the most occupationally exposed healthcare workers to ionizing radiation. Sources of exposure include direct radiation from patients who have received radiopharmaceuticals, contamination from handling radioactive materials, and scattered radiation in imaging suites, hot laboratories, and cyclotron rooms [2,3]. Sustained or excessive occupational exposure has been associated with potential long-term health risks, including increased cancer susceptibility and reproductive consequences, making radiation protection a critical priority.

The Kingdom of Saudi Arabia has witnessed substantial growth in nuclear medicine services over the past two decades, with facilities distributed across government, private, and military hospitals in all regions. Despite this expansion, concerns persist regarding the consistency of safety practices, training adequacy, equipment standards, and regulatory compliance across institutions [8].

Global nuclear medicine practice and the regional context

Nuclear medicine encompasses a wide range of diagnostic and therapeutic procedures, including myocardial perfusion imaging, bone scanning, dynamic renal imaging, 18F-fluorodeoxyglucose (18F) PET/CT (for oncology), and radioiodine thyroid imaging and therapy [1]. According to data compiled by the International Atomic Energy Agency (IAEA) in 2015, there were 910 gamma cameras across 17 regional countries, with 628 nuclear medicine centers. Saudi Arabia is among the five countries capable of assembling molybdenum-99/technetium-99m generator systems (99Mo/99mTc generators), with 1,157 nuclear medicine physicians, 1,953 technologists, 586 medical physicists, and 173 radiopharmacists serving the region [8].

Nuclear medicine staff are among the most heavily exposed healthcare workers to occupational ionizing radiation, with global average annual whole-body doses estimated at 1.9 millisieverts (mSv). Radiation dose is measured in mSVs per the United Nations Scientific Committee on the Effects of Atomic Radiation (UNSCEAR) [2,3]. In Saudi Arabia, Salama et al. confirmed doses within permissible limits among Eastern Province facilities, yet noted inconsistencies in protective practices [1].

Studies examining radiation safety awareness among healthcare workers consistently reveal significant knowledge gaps, including suboptimal awareness of dose limits and protection principles [7,9,11-14]. A nationwide survey in Guangdong Province, China (2016), found that rapid nuclear medicine expansion created potential risks for both workers and the public despite doses averaging 0.55 mSv. Radiation dose is measured (mSv) annually [9]. These findings collectively highlight the need for systematic, evidence-based assessment of nuclear medicine safety practices in rapidly growing healthcare systems such as Saudi Arabia.

Study objectives

This study aimed to (1) determine the practical effects and challenges in nuclear medicine as perceived by health professionals across Saudi Arabia; (2) assess the knowledge and practices of nuclear medicine professionals regarding radiation safety; (3) evaluate occupational complications and radiation exposure doses; and (4) identify key institutional challenges impeding safe nuclear medicine practice.

## Materials and methods

Study design

This was a cross-sectional, survey-based study designed to assess the knowledge, perceptions, occupational exposure, and challenges related to radiation safety among nuclear medicine professionals across Saudi Arabia. This study is reported in accordance with the Strengthening the Reporting of Observational Studies in Epidemiology (STROBE) guidelines for cross-sectional studies.

Study setting and period

The study was conducted across nuclear medicine departments in government, private, and military hospitals in all five geographic regions of Saudi Arabia (Central, Eastern, Western, Southern, and Northern regions). Data collection was conducted between May 2025 and June 2025 following ethical approval from the University of Bisha Research Ethics Committee, Bisha, Saudi Arabia (approval number: Ref No: UB-RELOC H-06-BH-087).

Study population and sampling

The target population comprised all licensed nuclear medicine professionals currently practicing in Saudi Arabia, including physicians/radiologists, medical physicists, radiopharmacists, nuclear medicine technicians, nuclear medicine nurses, and trainees. A convenience sampling approach was used, with the questionnaire distributed electronically via Google Forms (Google LLC, Mountain View, CA, USA) through professional WhatsApp groups (Meta Platforms, Inc., Menlo Park, CA, USA), professional networks, direct outreach to nuclear medicine departments, and social media platforms targeting nuclear medicine professionals across Saudi Arabia.

Eligibility criteria

Inclusion criteria were as follows: male and female healthcare professionals currently working or training in a nuclear medicine department in Saudi Arabia; roles including nuclear medicine physician/radiologist, medical physicist, radiopharmacist, nuclear medicine technician, nuclear medicine nurse, or trainee; and willingness to participate with completed informed electronic consent. Exclusion criteria were as follows: healthcare professionals not directly involved in nuclear medicine practice and incomplete questionnaire responses (less than 80% completion).

Sample size

Sample size was calculated using the Raosoft Sample Size Calculator (Raosoft, Inc., Seattle, WA, USA) with a 95% confidence interval and a 5% margin of error, yielding a minimum required sample size of 100 participants. A total of 110 completed responses were obtained.

Data collection instrument

A structured, self-administered questionnaire was developed after reviewing relevant literature on radiation safety, occupational exposure, and challenges in nuclear medicine practice. The questionnaire was designed in consultation with academic faculty members, expert consultants in nuclear medicine, community medicine specialists, and practicing nuclear medicine professionals to ensure content relevance and clarity (Appendix A).

Prior to the main study, a pilot study was 10 participants conducted in April 2025 among a small group of nuclear medicine professionals to evaluate the clarity, comprehensibility, and feasibility of the questionnaire items. Based on participant feedback, minor modifications were made before distributing the final version nationwide.

The questionnaire was distributed electronically via Google Forms and comprised five domains, namely, (1) demographic and professional information; (2) radiation knowledge and training; (3) safety perceptions and practices; (4) occupational exposure; and (5) desired future improvements.

Statistical analysis

Data were analyzed using IBM SPSS Statistics software, version 26.0 (IBM Corp., Armonk, NY, USA). Descriptive statistics summarized categorical variables as frequencies and percentages. Also used in the data analysis are both qualitative and quantitative data analysis methods. The data were organized using a Microsoft Excel sheet (Microsoft Corp., Redmond, WA, USA). Missing monitoring data were excluded from the denominator for those specific items and reported transparently.

Ethical considerations

Ethical approval was granted by the Research Ethics Committee of the University of Bisha (Reference No.: UB-RELOC H-06-BH-087, approved 21 May 2025). All participants provided informed electronic consent. Participation was voluntary, anonymous, and could be withdrawn at any time.

## Results

Participant characteristics

A total of 110 nuclear medicine professionals completed the survey, including 76 (69.1%) males and 34 (30.9%) females; 108 (98.2%) were Saudi nationals. The Central region provided the largest share of respondents (37.3%), followed by the Southern (21.8%), Eastern (20.0%), Western (11.8%), and Northern (9.1%) regions. Technicians (32.7%) and physicians/radiologists (28.2%) comprised the largest professional groups. Regarding experience, 38.2% had over 10 years in the field, while 20.9% were newly employed (≤1 year). Full demographic data are presented in Table 1.

**Table 1:** Demographic and professional characteristics of the participants (n=110).

Characteristic	Frequency (n)	Percentage (%)
Gender		
Male	76	69.1
Female	34	30.9
Age (years)		
20–24	8	7.3
25–29	17	15.5
30–34	21	19.1
35–39	24	21.8
40–49	29	26.4
≥50	11	10.0
Nationality		
Saudi	108	98.2
Non-Saudi	2	1.8
Region		
Central	41	37.3
Southern	24	21.8
Eastern	22	20.0
Western	13	11.8
Northern	10	9.1
Professional Role*		
Nuclear medicine (NM) technician	36	32.7
NM physician/radiologist	31	28.2
Radiopharmacist	19	17.3
NM nurse	19	17.3
Trainee	10	9.1
Medical physicist	9	8.2
*Multiple roles allowed; % may exceed 100%		
Years of experience		
≤1 year	23	20.9
2–5 years	19	17.3
6–9 years	26	23.6
10–15 years	22	20.0
>15 years	20	18.2
Sector*		
Government	64	58.2
Private	42	38.2
Military	11	10.0
Other	3	2.7
*Multiple sectors allowed; % may exceed 100%		
Total respondents	110	100.0

Working hours and stress levels

**Figure 1:**
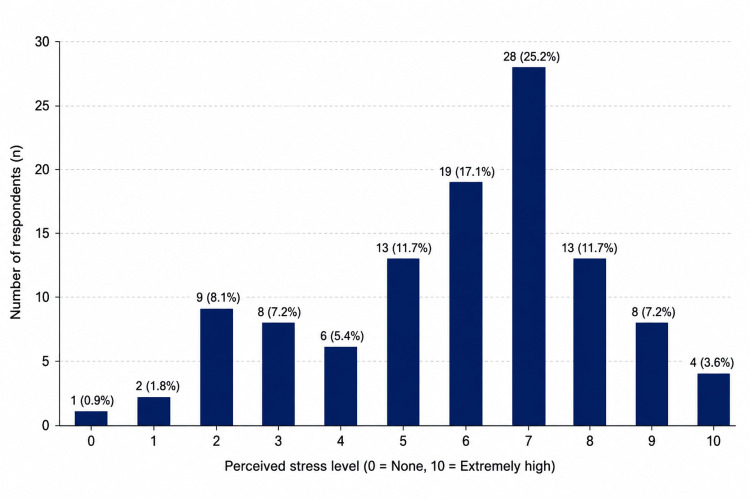
Distribution of perceived stress level at work.

The majority of respondents (77.3%; n=85) worked 40-50 hours per week, and 7.3% (n=8) exceeded 50 hours weekly (Table 2). Regarding occupational stress, 47.3% reported high stress levels (scores 7-10 on a 0-10 scale), as illustrated in Figure 1.

**Table 2:** Average number of hours worked per week.

Working Hours per Week	Frequency (n)	Percentage (%)
Less than 40 hours	17	15.5
40–50 hours	85	77.3
More than 50 hours	8	7.3
Total	110	100.0

**Figure 2:**
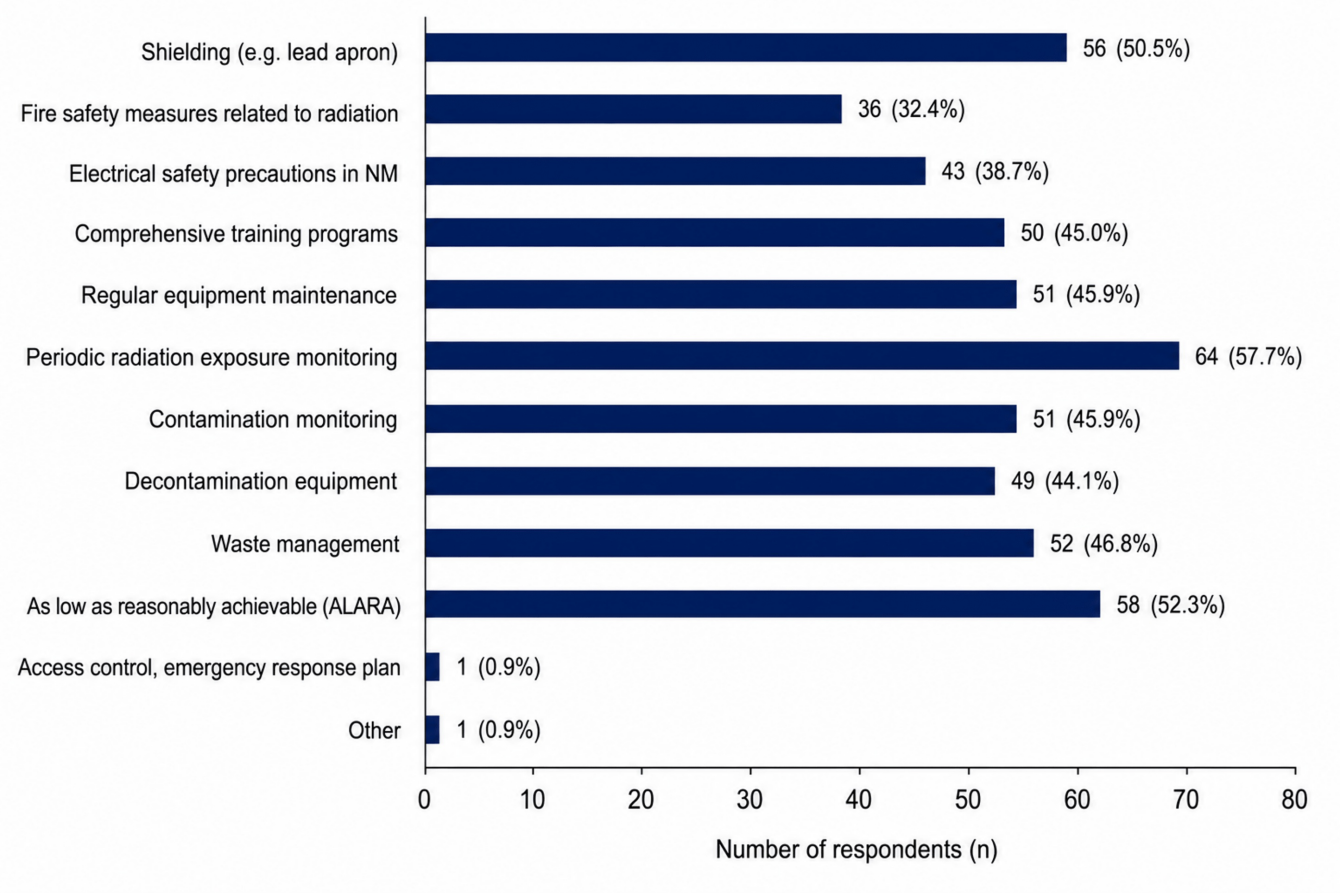
Distribution of stress-related factors among among nuclear medicine (NM) professionals.

The distribution of stress-related factors among participants is illustrated in Figure 2. 

Radiation knowledge

When self-rating knowledge of radiation risks, 24.5% (n=27) rated it as "Excellent" and 45.5% (n=50) as "Good." However, 26.4% (n=29) rated it "Fair" and 3.6% (n=4) "Poor," indicating approximately 30% may have suboptimal radiation knowledge (Table 3).

**Table 3:** Self-rated knowledge of radiation risks and long-term health effects.

Knowledge Rating	Frequency (n)	Percentage (%)
Excellent	27	24.5
Good	50	45.5
Fair	29	26.4
Poor	4	3.6
Total	110	100.0

Radiation safety perceptions

Only 45.5% of respondents agreed or strongly agreed that they were familiar with dose limits, while 25.5% disagreed. Training sufficiency was rated positively by 51.8% but negatively by 22.8%. Access to protective equipment was adequate for 71.8%, and 65.5% rated regular safety training as effective (Table 4).

**Table 4:** Radiation safety perceptions among nuclear medicine (NM) professionals (n=110).

Statement	Strongly Disagree	Disagree	Neutral	Agree	Strongly Agree
Familiar with dose limits	9.1% (10)	16.4% (18)	29.1% (32)	26.4% (29)	19.1% (21)
Sufficient training on NM protocols	6.4% (7)	16.4% (18)	25.5% (28)	30.9% (34)	20.9% (23)
Easy access to protective equipment	4.5% (5)	8.2% (9)	15.5% (17)	40.9% (45)	30.9% (34)
Regular safety training is effective	4.5% (5)	10.9% (12)	19.1% (21)	40.0% (44)	25.5% (28)

**Figure 3:**
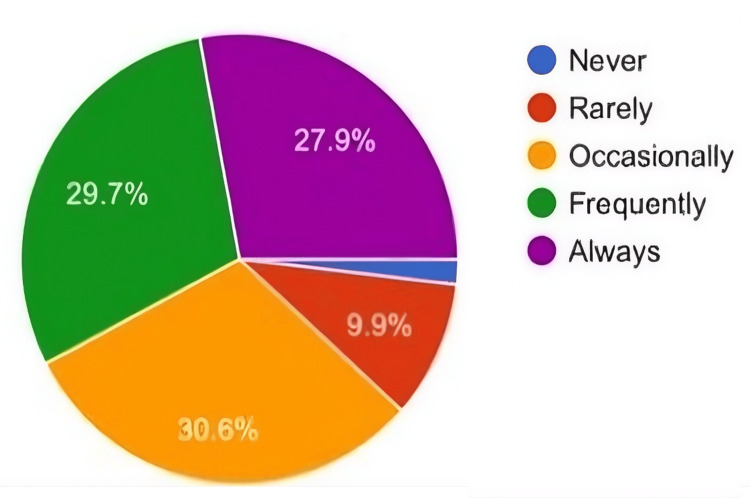
Effective radiation safety measures identified by nuclear medicine professionals and frequency of applying time, distance, and shielding strategies.

Effective radiation safety measures identified by nuclear medicine professionals and the frequency of applying time, distance, and shielding strategies are illustrated in Figure 3.

**Figure 4:**
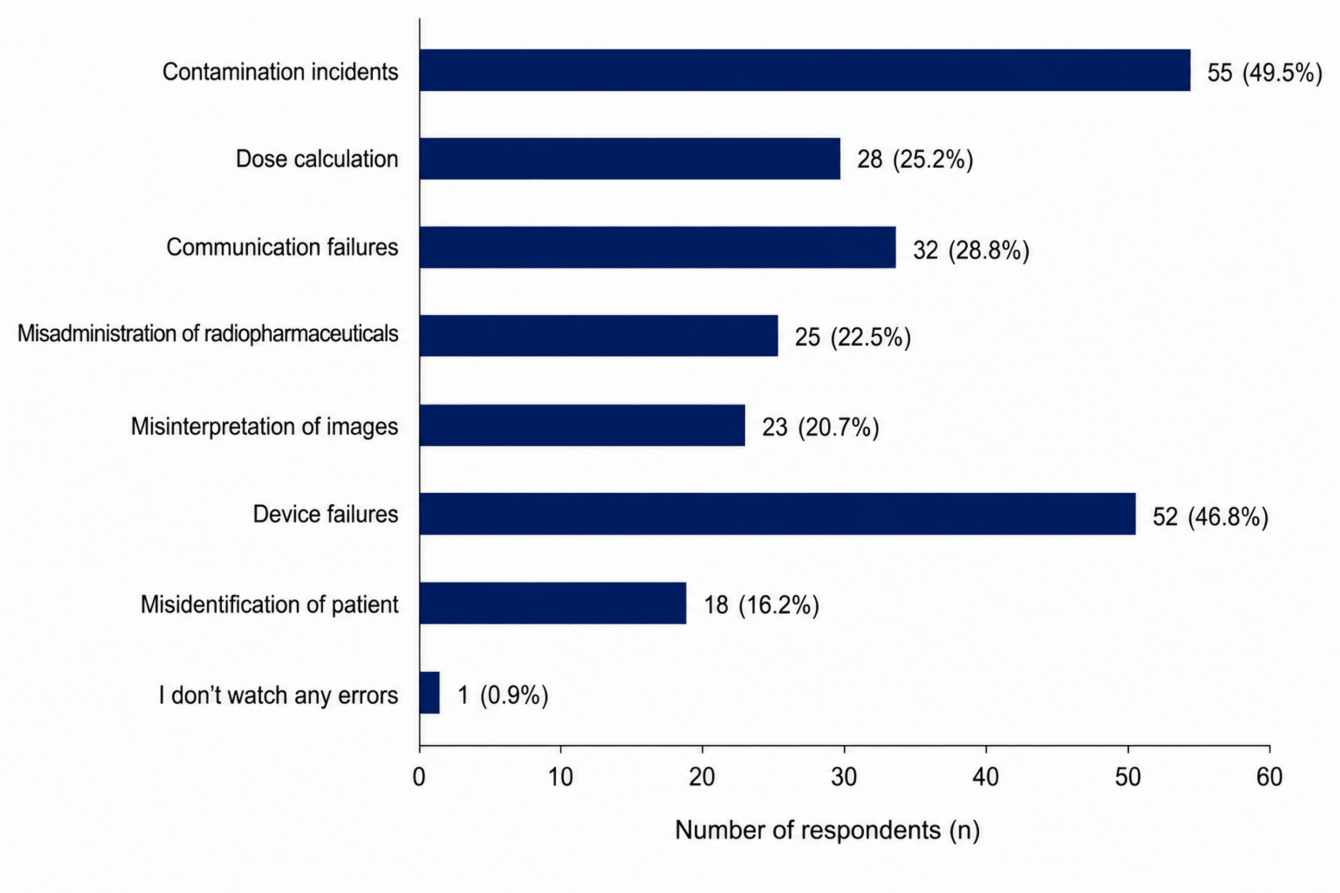
Role-specific error patterns durning practice

Figure 4 illustrates the role-specific distribution of the most common errors in nuclear medicine among healthcare professionals.

Radiation exposure data

Personal monitoring data were available for 84 of 110 respondents (76.4%) for annual exposure. Of those monitored, 50.9% (n=56) reported maximum annual exposures below 5 mSv; 20.0% (n=22) reported 5-10 mSv; and 5.5% (n=6) exceeded 10 mSv annually (Table 5). Quarterly thermoluminescent dosimeter (TLD) doses were below 1 mSv for 26.4% (n=29), 1-2 mSv for 45.5% (n=50), and above 2 mSv for 10.0% (n=11) (Table 6). Twenty-six respondents (23.6%) lacked personal monitoring data entirely.

**Table 5:** Average thermoluminescent dosimeter (TLD)-recorded dose over three months.

Average Quarterly TLD Dose (mSv)	Frequency (n)	Percentage (%)
Less than 1	29	26.4
1–2	50	45.5
More than 2	11	10.0
No monitoring device	20	18.2
Total	110	100.0

**Table 6:** Maximum radiation exposure recorded during the last year.

Maximum Annual Exposure (mSv)	Frequency (n)	Percentage (%)
Less than 5	56	50.9
5–10	22	20.0
More than 10	6	5.5
No monitoring device	26	23.6
Total	110	100.0

Errors, hazards, and complications

**Figure 5:**
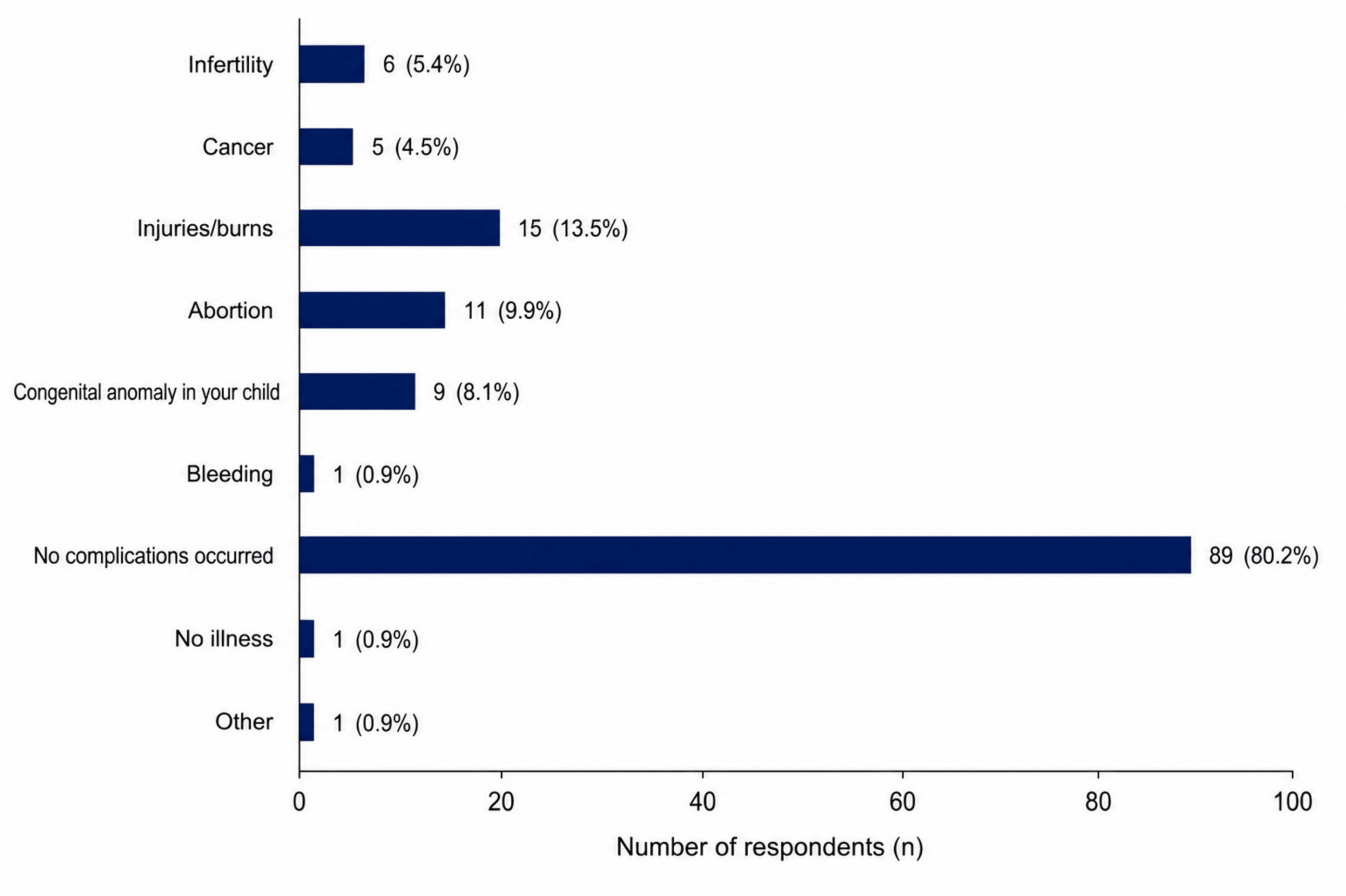
Most common errors in nuclear medicine stratified by professionals.

Role-specific error patterns are depicted in Figure 4. Radiation exposure was the predominant occupational hazard, followed by chemical and ergonomic hazards. Radiation-related complications experienced by respondents are summarized in Figure 5. 

**Figure 6:**
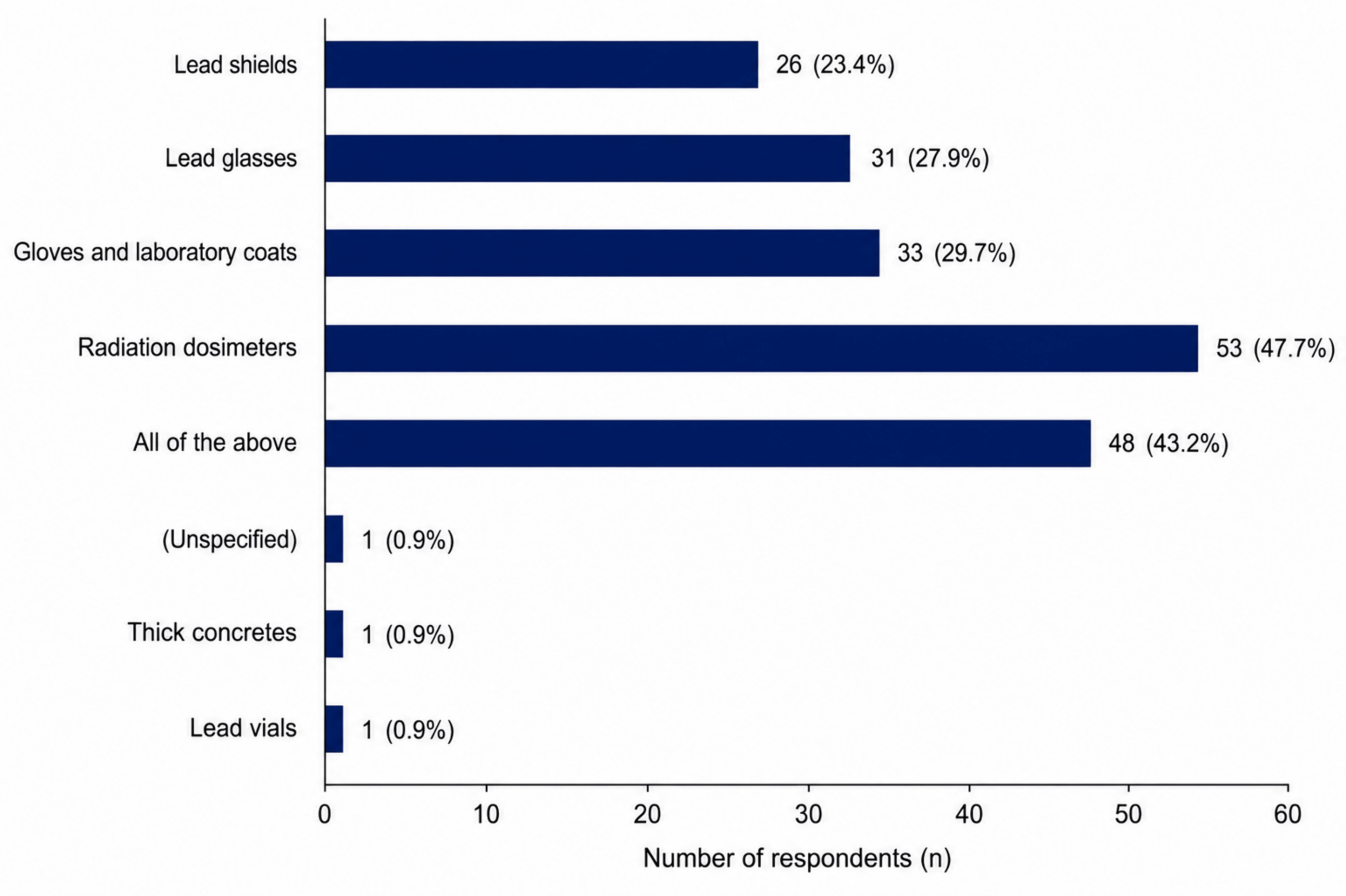
Radiation-related complications experienced by nuclear medicine professionals.

Protective equipment available in nuclear medicine centers is illustrated in Figure 6. 

**Figure 7:**
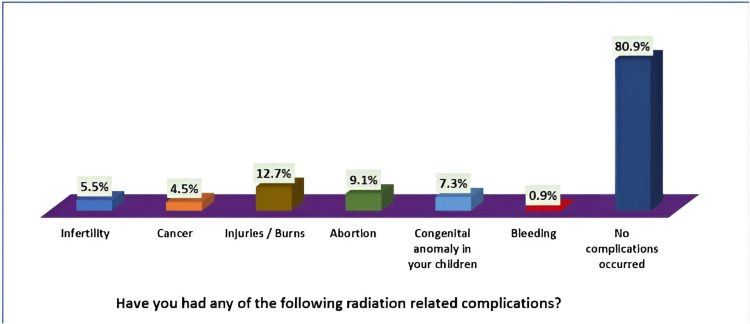
Radiation-related complications experienced by nuclear medicine professionals.

Participants reported experiencing the following radiation-related complications. This is illustrated in Figure 7.

The most frequently cited improvements were: expanding clinical nuclear medicine services (47.3%; n=52), enhancing safety measures and guidelines (42.7%; n=47), minimizing radiation exposure (40.0%; n=44), and providing continuous education and supervision (38.2%; n=42) (Table 7).

**Table 7:** Future improvements desired in nuclear medicine (NM).

Desired Improvement	Frequency (n)	Percentage (%)
Expanding clinical NM services across the Kingdom	52	47.3
Enhancing safety measures and guidelines	47	42.7
Minimizing radiation exposure	44	40.0
Expanding NM teaching facilities in health colleges	42	38.2
Providing continuous education, training, and supervision	42	38.2

**Figure 8:**
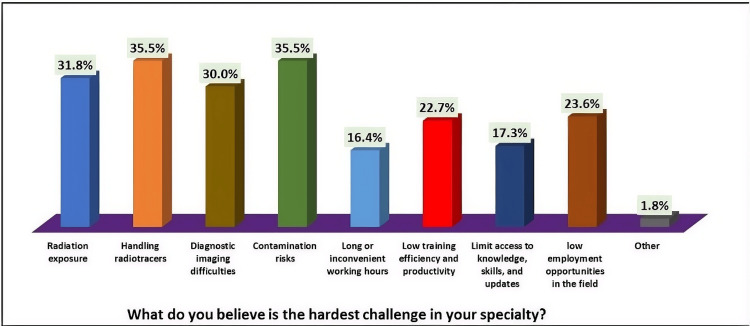
The hardest challenges faced by health professionals in nuclear medicine.

The perceived hardest challenges faced by health professionals in nuclear medicine are illustrated in Figure 8.

## Discussion

This cross-sectional study provides a nationally distributed assessment of radiation safety knowledge, perceptions, and occupational exposure among nuclear medicine professionals across all five regions of Saudi Arabia. The findings reinforce international evidence on occupational risks in nuclear medicine while highlighting context-specific challenges relevant to the Saudi healthcare system.

Knowledge and training gaps

Although 70% of respondents expressed moderate to high confidence in their radiation knowledge, only 45.5% were familiar with official dose limits, and 22.8% acknowledged insufficient training, a disconnect between perceived and actual preparedness documented in similar settings globally [6,12,14]. Salama et al. (2016) noted analogous inconsistencies among Saudi healthcare workers despite doses remaining within permissible limits [1]. Application of the time, distance, and shielding (TDS) triad was inconsistent across respondents (Figure 3). Training programs should be reformed to make TDS adherence a mandatory, auditable standard rather than a recommended practice.

Occupational exposure

The global average annual whole-body occupational dose for nuclear medicine workers is approximately 1.9 mSv, according to UNSCEAR [2]. In the current study, among the 84 respondents with available monitoring data, 5.5% (n=6) reported maximum annual exposures exceeding 10 mSv, and 10.0% (n=11) reported quarterly TLD doses above 2 mSv. Notably, 23.6% of respondents lacked personal monitoring data entirely, suggesting incomplete implementation of occupational radiation monitoring programs, a regulatory concern, given that individual dosimetry is fundamental to As Low As Reasonably Achievable (ALARA) compliance [3,7,11,13]. International data indicate that newer, less experienced staff contribute disproportionately to high-dose readings, [3] consistent with the 20.9% of respondents with one year or less of experience in this study.

Equipment and infrastructure

A proportion of respondents identified equipment-related challenges, including inconsistent access to shielding materials, as significant barriers to safe nuclear medicine practice. These infrastructure gaps are consistent with regional assessments highlighting disparities in nuclear medicine equipment availability between urban centers and peripheral hospitals [3,8]. ALMisned et al. (2021) demonstrated that bismuth-silicate glass composites offer superior gamma-ray attenuation compared with conventional lead barriers across common nuclear medicine radioisotope energies, presenting a viable pathway to improved protection [7].

Waste management and protocol adherence

Approximately 28.2% of respondents reported that radiation safety protocols were not consistently followed in their departments, and nearly half expressed uncertainty about waste management procedures. Ravichandran et al. (2011) emphasized the critical importance of structured radioactive waste handling protocols [2]. The gap between policy and implementation suggests that departmental audits and enforcement mechanisms are needed to reinforce compliance.

Occupational stress and workforce well-being

Nearly half of the respondents (47.3%) reported high occupational stress levels. High stress has been associated with increased procedural errors and protocol deviations, further linking workforce well-being to patient and occupational safety. The high proportion of workers working 40-50 hours weekly, with 7.3% exceeding 50 hours, suggests staffing levels may be insufficient relative to workload demands in some facilities.

Limitations

Several limitations should be acknowledged. First, convenience sampling may limit generalizability to all nuclear medicine professionals in Saudi Arabia. Second, the cross-sectional design precludes causal inference; associations should be interpreted as hypothesis-generating. Third, self-reported data are subject to recall and social desirability biases. Fourth, the absence of personal monitoring data for 23.6% of participants limits the completeness of exposure assessment. Fifth, the questionnaire was reviewed by expert consultants and pilot-tested among nuclear medicine professionals prior to distribution; however, formal psychometric validation (e.g., reliability and construct validity testing) was not performed. Future studies should employ validated instruments and longitudinal designs to further strengthen the evidence base.

The use of convenience sampling through social media platforms may have introduced selection bias and limited the generalizability of the findings, as digitally connected professionals may have been overrepresented.

Given the cross-sectional design and reliance on self-reported data, the findings should be interpreted with caution and cannot establish causal relationships. However, they provide valuable baseline evidence regarding radiation safety practices and perceptions among nuclear medicine professionals in Saudi Arabia.

## Conclusions

This study provides the first nationally distributed cross-sectional assessment of radiation safety knowledge, perceptions, and occupational exposure among nuclear medicine professionals across all regions of Saudi Arabia. While a majority express confidence in radiation knowledge, significant and actionable gaps exist in dose-limit familiarity, training adequacy, personal monitoring coverage, and adherence to radiation protection strategies. A notable proportion exceeded recommended exposure thresholds, and infrastructure challenges persist across facilities. A multi-pronged national response is needed, including (1) standardized, competency-based radiation safety training mandated for all nuclear medicine roles; (2) universal implementation of personal dosimetry programs across all facilities; (3) regular departmental safety audits and ALARA program reviews; (4) targeted investment in nuclear medicine equipment modernization and shielding infrastructure; and (5) expansion of nuclear medicine training programs in Saudi health colleges to address the current workforce shortage. Addressing these gaps is essential to ensuring the sustainable, safe, and effective growth of nuclear medicine in Saudi Arabia.

The rapid expansion of nuclear medicine services in Saudi Arabia should be accompanied by stronger radiation safety measures. Based on our findings, we recommend implementing standardized national radiation safety training programs, enhancing awareness of occupational dose limits through continuous education and periodic assessments, enforcing routine safety audits and mandatory personal dosimetry, ensuring equitable access to protective equipment and modern monitoring systems, and promoting interdisciplinary collaboration to strengthen the culture of radiation protection and improve workforce well-being. Collaboration with regulatory authorities and hospital management is recommended to translate these findings into practical policies, regular safety audits, and continuous quality improvement initiatives in nuclear medicine departments.

## Appendices

Appendix A

Consent 

Dear participant, thank you for your valuable time.

You are invited to participate in this questionnaire. The purpose of this research is to assess hazards and complications among nuclear medicine providers, their knowledge of safety measures regarding equipment, devices, radionuclides, and radiation protection practices, as well as to explore occupational radiation exposure doses and frequency among providers. Additionally, the study aims to identify risks and suggest strategies to help minimize hazards in the future.

Confidentiality of participants will be maintained. Participation in this study is voluntary, and you have the right to refuse or accept participation. Please complete the questionnaire if you agree to participate.

Thank you

Do you consent to participate in this questionnaire?

◯ Yes ◯ No

Questionnaire: Self-Administered (English Version)

Demographic information

1. Gender:

◯ Male

◯ Female

2. Nationality:

◯ Saudi

◯ Non-Saudi

3. In which regions of Saudi Arabia does your location center cover?

◯ Eastern region

◯ Western region

◯ Central region

◯ Southern region

◯ Northern region

How old are you (years)?

◯ 20-24

◯ 25-29

◯ 30-34

◯ 35-39

◯ 40-49

◯ More than 50

What is your role in a nuclear medicine facility? (Check all that apply):

▢ Nuclear medicine physician/radiologist

▢ Medical physicist

▢ Radiopharmacist

▢ Nuclear medicine technician

▢ Nuclear medicine nurse

▢ Trainee

▢ Other

What type of training program did you have in nuclear medicine?

▢ Radiology + Nuclear medicine

▢ Saudi Board of Nuclear Medicine

▢ Internship in nuclear medicine

▢ Other

How long have you been working in nuclear medicine?

◯ 1 year or less

◯ 2 - 5 years

◯ 6 - 9 years

◯ 10 - 15 years

◯ More than 15 years

8. Which sector do you work in? (Check all that apply):

▢ Medical government sector

▢ Private sector

▢ Military sector

▢ Other

9. What is the average number of hours you work per week? (Write your answer)

10. How do you rate the stress level in your work?

◯ 0 (No stress)

◯ 1

◯ 2

◯ 3

◯ 4

◯ 5

◯ 6

◯ 7

◯ 8

◯ 9

◯ 10 (Very stressful)

11. How do you rate your knowledge of risks and long-term health effects of radiation exposure?

◯ Excellent

◯ Good

◯ Fair

◯ Poor

12. You are familiar with the dose limits for health professionals working in the nuclear medicine department.

◯ Strongly disagree

◯ Disagree

◯ Neutral

◯ Agree

◯ Strongly agree

13. I have received sufficient training on nuclear medicine guidelines and protocols in nuclear medicine.

◯ Strongly disagree

◯ Disagree

◯ Neutral

◯ Agree

◯ Strongly agree

14. I am confident in following the guidelines and protocols of nuclear medicine.

◯ Not confident at all

◯ Not confident

◯ Neutral

◯ Confident

◯ Very confident

Risk knowledge (Perception)

15. How effective do you think the radiation protection measures in your hospital are in reducing radiation exposure risks?

◯ Ineffective

◯ Slightly effective

◯ Neutral

◯ Moderately effective

◯ Very effective

16. Which of the following radiation safety protection measures are useful against radiation hazards? (Check all that apply):

▢ Shielding (e.g., lead apron)

▢ Fire safety measures related to radiation areas

▢ Electrical safety precautions in radiation zones

▢ Regular maintenance and calibration of equipment

▢ Comprehensive training programs

▢ Regular equipment maintenance and calibration

▢ Periodic radiation exposure monitoring

▢ Contamination monitoring

▢ Decontamination equipment

▢ Waste management

▢ Keeping exposure As Low As Reasonably Achievable (ALARA) principle

17. Do you believe that radiation safety protocols in your department are strictly followed by healthcare professionals?

◯ Yes

◯ No

◯ Sometimes

18. How often do you use time, distance, and radiation protection strategies during procedures?

◯ Never

◯ Rarely

◯ Occasionally

◯ Frequently

◯ Always

19. I have easy access to radiation protection equipment (e.g., lead aprons, protective shielding).

◯ Strongly disagree

◯ Disagree

◯ Neutral

◯ Agree

◯ Strongly agree

20. Do you think regular training in radiation safety is effective for health professionals in nuclear medicine?

◯ Strongly disagree

◯ Disagree

◯ Neutral

◯ Agree

◯ Strongly agree

21. Is your current level of radiation protection knowledge sufficient?

◯ Highly insufficient

◯ Insufficient

◯ Neutral

◯ Sufficient

◯ Highly sufficient

22. What level of challenges do you face when working with nuclear medicine devices?

◯ No challenges

◯ Minor challenges

◯ Moderate challenges

◯ Significant challenges

◯ Major challenges

23. What’s the maximum radiation exposure you've recorded during the last year? (Write your answer)

24. What’s the average radiation dose you were exposed to during a period of 3 months, as recorded by TLD from the personal monitoring device? (Write your answer)

25. How often do health professionals experience radioactive contamination while handling radionuclide therapies in your facility?

◯ Always

◯ Often

◯ Rarely

◯ Never

◯ Not applicable (No radioactive therapy in this facility)

26. According to your role, what do you think are the most common errors that happen in nuclear medicine? (Check all that apply):

▢ Contamination incidents

▢ Dose calculation

▢ Communication failures

▢ Equipment malfunction

▢ Misadministration of radiopharmaceuticals

▢ Misinterpretation of images

▢ Device failure

▢ Misidentification of the patient

▢ Other

27. How often do malfunctions in equipment lead to safety concerns or complications?

◯ Always

◯ Often

◯ Sometimes

◯ Rarely

28. According to your role, what are the most common hazards you face in nuclear medicine practice? (Check all that apply):

▢ Radiation exposure

▢ Chemical exposure

▢ Ergonomic hazards (e.g., Workplace-related physical discomfort)

▢ Other

29. From your knowledge, where would be the most exposure to the radiation? (Check all that apply):

▢ Hot lab

▢ PET/CT rooms

▢ SPECT/CT rooms

▢ Cyclotron

30. According to your role, where do you believe the highest radiation levels are present in your workplace? (Check all that apply):

▢ Imaging room (PET/CT or SPECT/CT)

▢ Waiting room

▢ Preparation area/Hot lab

▢ Injection room

▢ Cyclotron room (if applicable)

▢ Other

31. What is the protective equipment in your center? (Check all that apply):

▢ Lead shields

▢ Lead glasses

▢ Gloves and laboratory coats

▢ Radiation dosimeters

▢ All of the above

▢ Other

32. How would you rate the overall safety culture in your workplace regarding nuclear medicine?

◯ Excellent

◯ Good

◯ Fair

◯ Poor

33. Have you had any of the following radiation-related complications? (Check all that apply):

▢ Infertility

▢ Cancer

▢ Injuries/Burns

▢ Abortion

▢ Congenital anomaly in your children

▢ Bleeding

▢ No complications occurred

▢ Other

34. What do you believe is the hardest challenge in your speciality? (Check all that apply)

▢ Radiation exposure

▢ Handling radiotracers

▢ Diagnostic imaging difficulties

▢ Contamination risks

▢ Long or inconvenient working hours

▢ Low training efficiency and productivity

▢ Limit access to knowledge, skills, and updates

▢ Low employment opportunities in the field

▢ Other

35. What changes or improvements would you like to see in the field of nuclear medicine in the future? (Check all that apply):

▢ Expanding clinical nuclear medicine services across the Kingdom

▢ Expanding or adding more facilities for teaching nuclear medicine in Saudi health colleges

▢ Enhance safety measures and guidelines

▢ Minimize radiation exposure

▢ Providing continuous education, training, and supervision

▢ Other
